# The adaptability of red blood cells

**DOI:** 10.1186/1475-2840-12-63

**Published:** 2013-04-11

**Authors:** Etheresia Pretorius

**Affiliations:** 1 Department of Physiology, Faculty of Health Sciences, University of Pretoria, Private Bag x323, Arcadia 0007, South Africa

**Keywords:** Red blood cells, Iron, Glucose, Diabetes, Hemochromatosis, Scanning electron microscopy

## Abstract

The most important function of red blood cells (RBCs) is the carrying of oxygen, but they are also involved in inflammatory processes and during coagulation. RBCs are extremely deformable and elastic, as they are exposed to shear forces as they travel through the vascular system. In inflammatory conditions, and in the presence of hydroxyl radicals, RBCs loose their discoid shape. Here, ultrastructure of RBCs is studied using a scanning electron microscope, and we determine how fast changes in healthy individuals are noted after exposure to iron and glucose. We compare shape changes in these experiments to RBCs from diabetic and hemochromatosis patients (wild type, as well as hereditary hemochromatosis with mutations H63D/H63D, C282Y/C282Y, H63D/C282Y, C282Y/wild type and H63D/wild type). Thrombin is also added to whole blood exposed to iron, glucose and blood from diabetes and hemochromatosis patients. RBCs are easily deformed to a pointed shape in smears, and, with the addition of thrombin they are entrapped in the fibrin mesh of dense matted fibrin deposits. This entrapping causes severe shape changes due to the pressure of the fibrin onto the stressed cells. The most important observation of the current research is therefore how fast RBC can adapt in a changed environment and that the pressure of fibrin fibers may trap the RBC tightly in the resulting clot.

## Introduction

Red blood cells (RBCs) are discoid shape entities without a nucleus or mitochondria and their most important function is the carrying of oxygen to the cells of the body. They also play a fundamental role in the coagulation system and in inflammation [1], and has been used in biochemical studies to determine levels of antioxidants [2]. However, their role in inflammatory conditions is sometimes under-valued. Recently, Lopes del Almeida and co-workers pointed out that RBCs are not only a hemoglobin filled sacs, but are involved intimately in inflammation [1].

RBCs are extremely deformable and elastic, as they are exposed to shear forces as they travel through the vascular system [3]. These functions are highly dependent on membrane composition, and it is this composition that defines the properties of the RBC. Their membranes consist of 3 layers, the carbohydrate-rich glycocalyx on the exterior, the lipid bilayer that contains trans-membrane proteins and lastly, the membrane skeleton consisting of a structural network of proteins located on the inner surface of the lipid bilayer. In particular, the proteins of the membrane skeleton are responsible for the deformability, flexibility and durability and aids in recovering the discoid shape during rheology. The roughness of the cell membrane is also an indicator of cell's health state [4,5] and the skeletal integrity of the membrane, measured as surface roughness, is well correlated to the functional status of the cell, with a decrease of the membrane roughness seen in cells from diseased individuals [3,6]. Recently, researchers also reported that RBC distribution width (which is a measurement of the size variation as well as an index of the heterogeneity), is an easy, inexpensive, routinely reported test, and that may be used as significant diagnostic and prognostic information in patients with cardiovascular and thrombotic disorders [7].

The rheological characteristics of erythrocytes are therefore closely associated with membrane structure, and can be negatively influenced by different factors including the concentration of cholesterol, fibrinogen and gamma-globulins found in the plasma. Factors connected with the structural conformation of the cell membrane, as well as the intracellular levels of ATP, which are inversely proportional to the cytosolic concentration of calcium, also impair the rheology of the RBCs.

As mentioned previously, the oxygen carrying capacity is the function that RBCs are probably most associated with. Although iron plays is an important role this process, as it facilitates the carrying of oxygen, iron overload may be detrimental, not only to the RBCs, but also to all tissues in the body [8]. Therefore iron is rigorously regulated because excessive iron causes damage due to the formation of hydroxyl radicals [9].

Iron overload is implicated in many diseases [10], and probably the most well-known is hemochromatosis, where serum iron levels may exceed 10× the normal values compared to healthy individuals. Hereditary hemochromatosis is a genetic iron overload condition that can lead to unregulated absorption of iron [11]. Except for this condition, iron is also implicated as a major cause of diabetes [12-14]. Diabetes also frequently occurs in hemochromatosis patients [15]. Interestingly, the pathophysiology of diabetes in hemochromatosis is thought to be due primarily to defects in the early insulin response to glucose [11]. This relationship was also found where higher serum ferritin level and higher heme iron intake is associated with elevated risk of diabetes [16].

Recently, we have shown that in diabetes, the RBC membranes have a changed roughness when using atomic force microscopy (AFM) [5]. Measurement of surface roughness showed a decrease in roughness and alterations in the cytoskeletal matrix and the connections between band 3 and 4 proteins and the matrix. Furthermore, AFM measurement of the macro-parameters indicated that erythrocytes in diabetes are smaller, with a reduced concave depth. Also, a changed superficial protein structure rearrangement was noted [5].

Scanning electron microscopy (SEM) has also shown that in inflammatory diseases like thrombo-ischemic stroke and diabetes, RBCs have a changed ultrastructure [10,17]. Previously, we have also shown that exposing RBCs of healthy individuals to iron, can also induce similar shape changes [10]. The changes were ascribed to the formation of hydroxyl radicals in experiments and the presence of these radicals in diseases conditions that ultimately lead to inflammation [18]. The question that now arises is whether ultrastructure can give us insights on how fast these changes may occur; and ultimately therefore, how adaptable these cells are. We know that RBC shape changes in diabetes, and when physiological levels of iron are added to healthy individuals. Here we determine if such changes are also seen in hereditary hemochromatosis. Also, we investigate if addition of glucose to healthy whole blood will induce changes to these cells, and compare results to when iron is added to healthy whole blood. The addition of iron and glucose was done to determine how fast RBCs can adapt in the presence of a changed environment. Additionally, as it is known that the coagulation system is changed in the presence of iron overload, due to the generation hydroxyl radicals in particular, we also compare RBC shape changes in the presence of thrombin, that creates fibrin networks and dense matted deposits in inflammation [19]. Therefore, RBC changes, in the presence of iron and glucose with the addition of thrombin, as well as in diabetes and hemochromatosis (also with and without the addition of thrombin) were studied and compared to that of RBCs from healthy individuals.

## Materials and methods

### Human subjects

Ten non-smoking healthy individuals with no chronic diseases and who do not use any medication, were used as control subjects and compared to our SEM data base of thousands of micrographs, and found to be comparable. Diabetes and hemochromatosis patients (wild type, as well as hereditary hemochromatosis with mutations H63D/H63D, C282Y/C282Y, H63D/C282Y, C282Y/wild type and H63D/wild type) were identified by genetic testing. RBCs in diabetes have been studied the past 2 years in our lab and we also have such a RBC database. The current sample consisted of 10 diabetes patients, whose micrographs were also comparable to this database, and also found to be comparable. Twenty haemochromatosis patients were part of the current sample, with measured serum ferritin levels ranging between 350 and 1900 μg/L (0.001 to 0.007 mM). Currently a hemochromatosis database is being created.

### Preparation of blood samples

Whole blood samples from healthy individuals, individuals with diabetes and hemochromatosis were obtained in citrated blood tubes. To prepare whole blood smears, 10 μl aliquots were directly placed on a glass cover slip with and without the addition of 5 μl human thrombin (20 U/mL).

### Iron experiments

In experiments with iron, 5 μl of 0.03 and also 1.5 mM ferric chloride solution was mixed with 10 μl whole blood from healthy individuals on the glass cover slip and left for 3 minutes, followed with and without the addition of 5 μl thrombin. Final iron concentration is therefore 0.015 or 0.0075 mM where 0.03 mM was added and where 1.5 mM ferric iron was added the final iron concentrations in the experiment was 0.75 or 0.375 mM. The serum ferritin levels of the current hemochromatosis/iron overload group ranged between 350 to 1900 μg/L (tested in a pathology lab). Literature metioned that in iron overload the plasma NTBI levels are 0.002 to 0.01 mM [20,21]; and in Thalassemia up to 0.016 mM [22].

### Glucose experiments

10 μl whole blood from healthy individuals (fasting sugar levels of 5.2 mM/L) was mixed with 10 μl of 10 mM glucose after waiting for 3 minutes and followed with and without 5 μl human thrombin.

### Scanning Electron Microscopy (SEM) procedure

Before further processing the samples were incubated at room temperature for 10 minutes. After incubation the samples were immersed in 0.075 M sodium phosphate buffer, pH 7.4, and placed on a shaker for 20 minutes. The samples were then fixed in a 2.5% formaldehyde/glutaraldehyde solution after which standard SEM procedures were followed, including carbon coating. All samples were viewed with a ZEISS FEG scanning electron microscope at 1 kV.

## Results

**Figure 1:**
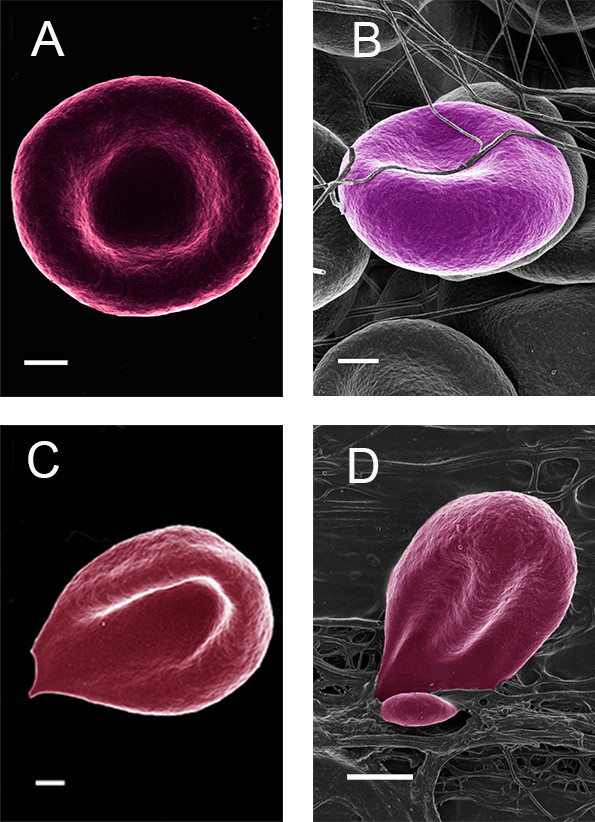
**(A) Healthy RBC (B) Healthy RBC with added thrombin; (C) Healthy RBC with added iron; (D) Healthy RBC with added iron and thrombin.** Scale = 1 μm.

Figure 1A and B shows an RBC from a healthy individual with and without the addition of thrombin. In a typical whole blood smear of healthy individuals, the RBCs are discoid in shape. With the addition of thrombin, fibrin fiber nets form around and over the RBCs. In general, in healthy individuals the RBCs do not change shape due to the pressure of the fibrin fibers laying over them (Figure 1B). Both 0.03 and 1.5 mM ferric chloride iron concentrations caused changes in RBC and RBC with thrombin. The addition of physiological levels of iron, caused RBCs to change to show a pointed extension (as preciously shown by our group); while the addition of thrombin caused the RBCs to further change shape and deform around the generated fibrin fibers (Figure 1C and D).

**Figure 2:**
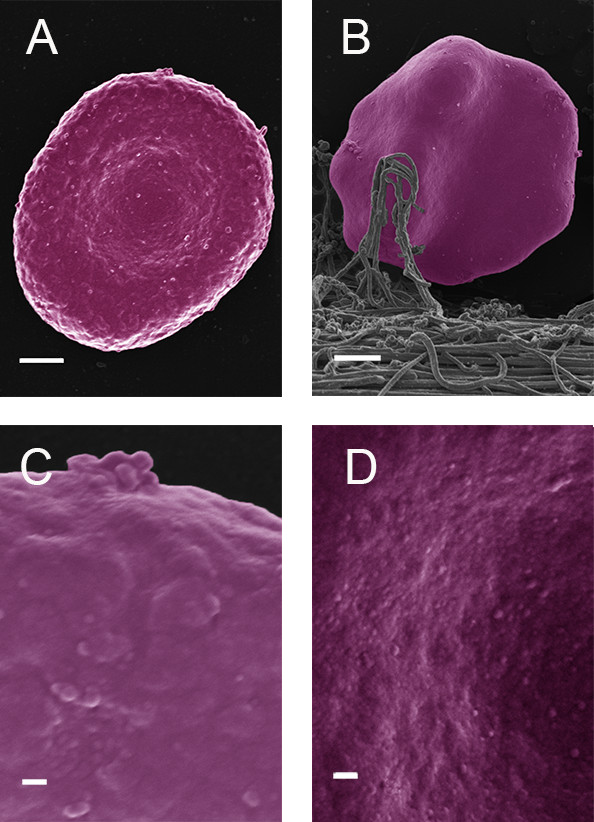
(A) Healthy RBC with added glucose (Scale = 1 μm); (B) Healthy RBC with added glucose and thrombin (Scale = 1 μm); (C) High magnification healthy RBC with added glucose (Scale = 100 nm); (D) High magnification healthy RBC (Scale = 100 nm).

When glucose is added to the whole blood from healthy individuals, only minor shape changes occur, where the cells still appear discoid, but slightly elongated (Figure 2A). With the addition of glucose, as well as thrombin, the RBCs also appeared to be more flexible and deform easily around the fibrin fibers (Figure 2B). Also, at very high machine magnification, the surface membrane structure is changed to a more bulbous ultrastructure (Figure 2C) compared to healthy RBCs (Figure 2D). This happens within the very short period of exposure to glucose.

**Figure 3:**
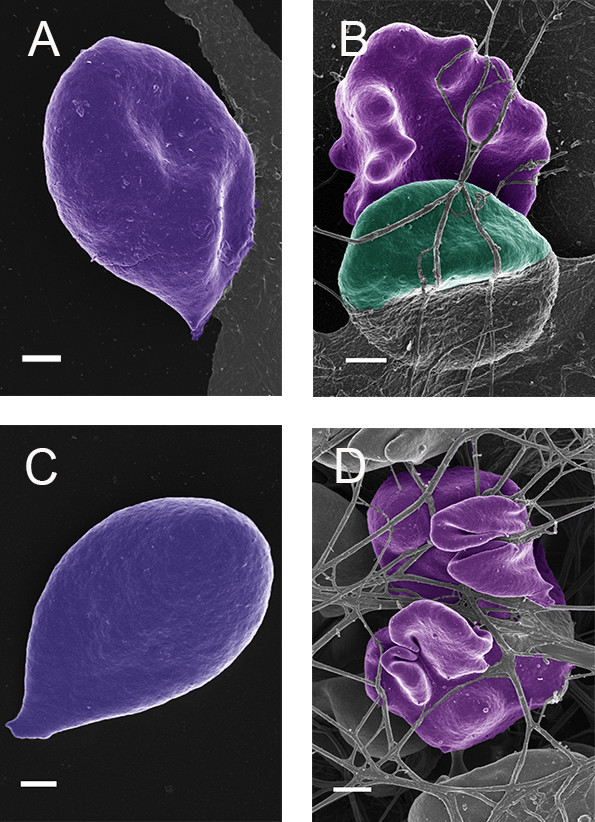
**(A) Diabetes RBC; (B) Diabetes RBC with added thrombin; (C) Hemochromatosis RBC; (D) Hemochromatosis RBC with added thrombin.** Scale = 1 μm.

In diabetes patients we have also seen an altered RBC morphology (Figure 3A) and with the addition of thrombin, the RBCs deform easily as the fibrin fibers press onto the RBCs (Figure 3B). This was also noted in hemochromatosis (Figure 3C and D).

## Discussion

In healthy individuals, RBC morphology show the typical discoid shape (Figure 1A) while the addition of thrombin, entraps the RBC within the mesh (Figure 1B). In healthy individuals, these cells deform insignificantly when fibrin is formed around and over it, and the typical discoid shape is still noted here. However, with the addition of iron, causing the production of hydroxyl radicals [23], a shape change occurs, where the RBCs are distorted to a pointed structure (Figure 1C). This has also previously been reported [10]. With the addition of thrombin (and iron), dense matted fibrin fibers are generated; and here we see the shape change not only to slightly deformed RBC shape as in Figure 1C, but a major twisting and giving away of the RBC architecture, as the fibers and deposits press onto and around the cells (Figure 1D). Therefore, it seems as if the deformations are much more prominent with the addition of thrombin together with iron. Here we suggest that it is due to the damage induced by hydroxyl radicals caused by iron, and that the radicals damage the membrane and cellular cytoskeletal arrangements to such an extent that it allows the cell to deform easily under the pressure of the fibrin fibers and deposits. This phenomenon is interesting, when it is taken into account that the whole blood is exposed to physiological levels comparable to iron overload and only to 3 minutes followed by incubation of 10 minutes before termination of activity.

In the current research, the adaptability of RBCs was also seen with the addition of glucose, at physiological levels comparable to that of high sugar levels (Figure 2A). However, glucose does not generate hydroxyl radicals, therefore, it was expected that we would not see a fundamental shape change as is noted when iron is added, where hydroxyl radicals are generated. Due to the architectural membrane changes as a result of glucose exposure, the RBCs also deformed easily with the addition of thrombin (Figure 2B).

Here we suggest that this capability to deform is possibly due to the loss of the ability to maintain the discoid shape. This loss of shape allows these RBCs to distort and collapse freely under the weight of the fibrin fibers pressing against then, entrapping them in the meshwork. The topographic membrane changes (as seen at very high machine magnifications of 100000× magnification), due to glucose, also confirm the extremely quick adaptation capabilities of RBCs (Figure 2C compared to 2D).

When comparing the results obtained under lab conditions, as discussed in the above paragraphs, to diabetes and hemochromatosis patients (wild type, as well as hereditary hemochromatosis with mutations H63D/H63D, C282Y/C282Y, H63D/C282Y, C282Y/wild type and H63D/wild type), the same morphological trends are observed. In both cases these patients have systemic inflammation as additional factor. Here we also show that the RBCs are easily misshapen to a pointed shape in smears, but, with the addition of thrombin, they are entrapped in the fibrin mesh and this entrapping causes severe shape changes due to the pressure of the fibrin and dense deposits onto the stressed cells. This is seen in Figure 3A to D which shows RBCs form diabetes as well as hemochromatosis patients.

Hypercoagulability with resulting thrombotic complications in diabetes is well-known and this entrapping of the RBCs may be an additional factor for tight clot formation in this condition. However, hypercoagulability in hemochromatosis has not frequently been described in the literature, and little is known about the effects of iron overload on hemostasis and platelet activation [24]. Human hemochromatosis protein (HFE), the protein incriminated in the pathogenesis of hemochromatosis, competes with transferrin for binding to the receptor, thereby impeding the uptake of iron from transferrin [25]. Mutation of HFE destroys this competition, thus facilitating access of transferrin and its iron to cells [25]. Unfortunately, very little research is available on the effect of hemochromatosis on RBCs and fibrin fiber generation. Here RBC changes in hemochromatosis are ascribed to abnormal serum ferritin levels, as changes were noted in both wild type and confirmed hereditary hemochromatosis patients. Patients with diabetes, in many cases, also have high serum iron levels.

The most important observation of the current research is therefore how fast RBC can adapt in a changed environment and that the pressure of fibrin fibers that might form in a thrombotic event or just during normal coagulation processes may trap the RBC in the resulting clot. This cannot be studied using a light microscope, as the changes are too small to view with the magnification potential of such an instrument. Furthermore, no other measure or biochemical analysis will give such information. From these observations it is concluded that SEM analysis, although one of the oldest techniques in modern science, may provide novel and important information in drug therapy and clinical trials. RBCs may be used as a general barometer of the health status of an individual and its ultrastructure should be an irreplaceable tool due to its adaptability.

### Ethical approval disclosure

Ethical approval was granted at the University of Pretoria (HUMAN ETHICS COMMITTEE: FACULTY OF HEALTH SCIENCES) under E Pretorius. All human blood samples obtained were analyzed at the University of Pretoria and all participants filled in informed consent forms.

## Competing interest

There is no competing interest for the author.
